# New Mode of Administering Nitrous Oxide Gas

**Published:** 1870-01

**Authors:** 


*New Mode of Administering Nitrous Oxide Gas.*—The *Lancet* informs us that some interesting improvements in the mode of administering nitrous oxide gas have recently been perfected by Mr. Coleman, at the Dental Hospital of London. On Thursday, the 17th inst., this gentleman anæsthetized upon a new plan eight patients in the hospital, for the removal of teeth. All who have had much experience in the use of the protoxide of nitrogen have found that any admixture of air with the gas greatly impairs its efficacy. The insensibility is less rapidly and less perfectly produced under these circumstances, whilst the patient is invariably more excited during, and more prostrated after, the inhalation of the gas. In order, therefore, to secure the entrance into the blood, through the lungs, of pure nitrous oxide without accompanying air, Mr. Coleman so arranges his apparatus that he first dilutes with nitrogen the oxygen of the residual air of the lungs, and then permits the patient to breathe pure nitrous oxide. By this method the patients become more speedily unconscious, and the anæsthesia lasts longer, at the same time that they appear throughout the adminstration calmer than is usually the case under the ordinary mode of administration. It is a noteworthy fact, also, that in none of the eight cases was there the least appearance of lividity. During the exhalation of the nitrogen, the pulse was noticed to fall steadily, but upon supplying the nitrous oxide it soon regained its force and frequency. Mr. Coleman stated to those who witnessed the inhalation, that he believed that hydrogen would be more succesful than nitrogen, but as the inhalation of that gas often produces disagreeable sensations, he preferred trying the effects of nitrogen before employing hydrogen. If, by this new mode of administration, it shall be found that the anæsthesia produced by nitrous oxide can be rendered more lasting, say for a period of even only ten or fifteen seconds, It will be a great boon to the dentists, who oftentimes have most uncomfortably to expedite their operations with it. As regards cost, the above plan would be found the most economical of any yet devised.

